# Pathogenic LRRK2 requires secondary factors to induce cellular toxicity

**DOI:** 10.1042/BSR20202225

**Published:** 2020-10-14

**Authors:** Evy Lobbestael, Chris Van den Haute, Francesca Macchi, Jean-Marc Taymans, Veerle Baekelandt

**Affiliations:** 1 Laboratory for Neurobiology and Gene Therapy, Department of Neurosciences, KU Leuven, Herestraat 49 bus 1023, 3000 Leuven, Belgium; 2 Instituto Italiano di Tecnologia, via Morego 30, 16163 Genova, Italy; 3 Université de Lille, Inserm, CHU Lille, UMR-S1172, LilNCog, Lille Neuroscience & Cognition, 59000 Lille, France; 4 Inserm, UMR-S 1172, Team "Brain Biology and Chemistry", 59000 Lille, France

**Keywords:** cellular assays, leucine rich repeat kinase, neurite outgrowth, Parkinson's disease, skein-like strucutres

## Abstract

Pathogenic mutations in the leucine-rich repeat kinase 2 (LRRK2) gene belong to the most common genetic causes of inherited Parkinson’s disease (PD) and variations in its locus increase the risk to develop sporadic PD. Extensive research efforts aimed at understanding how changes in the LRRK2 function result in molecular alterations that ultimately lead to PD. Cellular LRRK2-based models revealed several potential pathophysiological mechanisms including apoptotic cell death, LRRK2 protein accumulation and deficits in neurite outgrowth. However, highly variable outcomes between different cellular models have been reported. Here, we have investigated the effect of different experimental conditions, such as the use of different tags and gene transfer methods, in various cellular LRRK2 models. Readouts included cell death, sensitivity to oxidative stress, LRRK2 relocalization, α-synuclein aggregation and neurite outgrowth in cell culture, as well as neurite maintenance *in vivo*. We show that overexpression levels and/or the tag fused to LRRK2 affect the relocalization of LRRK2 to filamentous and skein-like structures. We found that overexpression of LRRK2 per se is not sufficient to induce cellular toxicity or to affect α-synuclein-induced toxicity and aggregate formation. Finally, neurite outgrowth/retraction experiments in cell lines and *in vivo* revealed that secondary, yet unknown, factors are required for the pathogenic LRRK2 effects on neurite length. Our findings stress the importance of technical and biological factors in LRRK2-induced cellular phenotypes and hence imply that conclusions based on these types of LRRK2-based assays should be interpreted with caution.

## Introduction

Mutations in leucine-rich repeat kinase 2 (LRRK2) are the most common cause of genetic forms of Parkinson’s disease (PD) known to date [1,2]. Moreover, association studies have identified variations in the LRRK2 locus as a risk factor for sporadic PD [3–6]. LRRK2 encodes a large multi-domain protein comprising two catalytic domains and several predicted protein–protein interaction domains [7]. Since its identification in 2004, intense investigations have focused on identifying the mechanisms by which pathogenic mutations in LRRK2 affect its physiological function(s) that lead to neurodegeneration. The ultimate goal was the development of a robust LRRK2-based model that recapitulates key features of PD pathology. Overexpression of pathogenic variants of LRRK2 in cell culture or primary neuronal cells has been reported to induce relevant phenotypes, including cell toxicity, increased sensitivity to oxidative stress, disturbed neurites and protein inclusion formation. However, these phenotypes could not always be replicated in independent studies [8].

Indeed, mutant LRRK2-induced cell toxicity has been replicated by several groups, albeit with large variability ranging from 10 to 70% cell death [8–13], while other reports have shown no effect on cell viability compared with wild-type (WT) LRRK2 or control conditions [8,14–17]. Another LRRK2-related phenotype observed in cell culture is the formation of LRRK2-containing inclusions. LRRK2 accumulations have been observed both in WT [12,18–24] and mutant LRRK2 overexpressing cells [12,25–30]. Roughly, two types of LRRK2 accumulations can be distinguished: aggregates or punctae and filamentous structures, or so-called skein-like structures [19,31]. Interestingly, skein-like structures are very prominent upon LRRK2 kinase inhibition [32–34] and were suggested to be regulated by GTP binding [31].

In addition, reduction of neurite length by mutant LRRK2 has been repeatedly observed in primary neurons from LRRK2 transgenic rodents [35–40] or in primary neurons [9–12,15,41,42] and cell lines [43–45]. However, the effect on neurite length appears highly dependent on the experimental setup [14,15,46,47]. Disturbed neurites have also been described *in vivo* after *in utero* electroporation of mutant LRRK2 in the cortex [28], dentate gyrus and substantia nigra of BAC LRRK2 G2019S [48] and R1441G transgenic mice [49], respectively. More recently, P21-activated kinase 6 (PAK6) has been identified as a LRRK2 interactor and regulator of neurite complexity. Interestingly, the G2019S LRRK2-associated neurite shortening in primary neurons could be rescued by a constitutively active form of PAK6 [50]. Moreover, LRRK2 was required for the increased neurite outgrowth in mouse striatum, induced by PAK6 overexpression [51].

Finally, given the prominent role of (aggregated) α-synuclein in PD patients, many studies have found indications that α-synuclein and LRRK2 are involved in the same pathological pathway, but probably not directly interacting [52–54]. Cell culture models have shown that (pathogenic) LRRK2 can enhance α-synuclein protein levels and inclusion formation [12,17,55–59].

Taken together, different LRRK2-based PD cell culture models have been proposed, each with their own advantages and relevance, but with considerable variability in the observed phenotype. Here, we aimed to identify possible critical parameters that can (partly) explain the observed outcome, and can be used to develop a standardized research protocol.

## Materials and methods

### DNA constructs and reagents

Eukaryotic expression constructs for α-synuclein, 3flag-LRRK2 or eGFP-LRRK2 overexpression are described elsewhere [34,60–63]. Untagged LRRK2 was generated by BamHI restriction-mediated excision of the 3flag-tag. LRRK2 knockdown constructs were cloned according to [64] and used with a blasticidin resistance marker (as in [34]). All transgenes were cloned in the pCHMWS backbone as described in [65]. A CMV promoter was used for all cell culture applications, while a CaMKII0.4 promoter was applied for *in vivo* overexpression purposes. All constructs were sequence confirmed. Lentiviral (LV) vectors were produced as described in Ibrahimi et al. [66]. Anti-α- or β-tubulin, vinculin and anti-FlagM2 antibodies are purchased from Sigma-Aldrich, anti-α-synuclein antibody from Enzo Life Sciences, LRRK2 P-S935 from Novus Biologicals and MJFF-2 from Abcam. Anti-eGFP antibody is generated in-house [67]. Terminal deoxynucleotidyl transferase dUTP nick end labeling (TUNEL) and lactate dehydrogenase (LDH)-based viability kits are purchased from Roche. LRRK2 kinase inhibitor-1 (L2-IN1) [32] was purchased from Calbiochem and PF-06447475 [68] from Sigma-Aldrich.

### Transgene transfer and cell lines

SH-SY5Y cells were maintained in DMEM (Thermofisher) medium supplemented with 15% heat-inactivated fetal calf serum (FCS, Thermofisher), 1% non-essential amino acids (Thermofisher) and 50 μg/ml gentamycin. All cells were maintained at 37°C in a humidified atmosphere containing 5% CO_2_ and were mycoplasma-free. Transfection of cells was performed with Fugene 6.0 (Promega) according to the manufacturer’s protocol. Cells were analyzed 48 h after transfection. Transduction was obtained by applying LV vectors to the cells for 48 h. Stable cell lines selected for LRRK2 knockdown or overexpression were generated by transduction of LV vectors encoding the transgene together with a blasticidin or hygromycin selection marker. Cells were maintained with the regular culture medium supplemented with 200 μg/ml hygromycin or 15 μg/ml blasticidin (InvivoGen). SH-SY5Y cells with overexpression of α-synuclein combined with LRRK2 overexpression or knockdown were generated in two steps to ensure equal α-synuclein expression in all conditions. First, cells were transduced with LV vectors encoding α-synuclein together with a puromycin selection marker. After selection of the cells for α-synuclein expression (1 μg/ml puromycin), cells were transduced with different LRRK2 vectors and selected for LRRK2 overexpression (hygromycin) or knock down (blasticidin). To differentiate SH-SY5Y cells, fresh medium with 5% FCS and supplemented with 10 μM retinoic acid (Sigma-Aldrich) was given 1 day after plating. This was repeated for 4 days, followed by fixation of the cells. In the condition of cell transduction at the end of the differentiation protocol, LV vectors were added to the medium 5 days after plating and cells were fixed 3 days after transduction. All cell lines are polyclonal.

### Cellular assays

The sensitivity of a cell line for oxidative stress was measured using an LDH Cytotoxicity Assay Kit (Sigma-Aldrich). Cells were plated in 96-well vessels and exposed to a dilution series of H_2_O_2_ (0–800 μM) for 16 h and release of LDH was measured according to the manufacturer’s protocol using the EnVision Multilabel Plate Reader (Perkin Elmer). The obtained values were used to fit a dose–response curve and calculate EC_50_ values. The level of apoptosis of transfected and transduced cells was investigated using the TUNEL assay kit (Sigma-Aldrich) according to the manufacturer’s instructions. By combining the TUNEL assay with immunocytochemistry for the transgene, only cells with transgene overexpression were taken into account. At least 100 cells per coverslip were counted.

For the LRRK2 localization experiments, SH-SY5Y cells were treated with compound or solvent for 2 h with 1 μM LRRK2-IN1 or 150 nM PF-06447475 without replacing the medium. The effect of LRRK2 on α-synuclein-induced toxicity and inclusions was investigated by introducing LRRK2 knockdown or overexpression in the synucleinopathy cell culture model as described in [63]. In short, cells were plated in a 96-well plate and treated with 100 μM H_2_O_2_ and 5 mM FeCl_2_ for 72 h. Fixed cells were stained with 0.1% Thioflavin S (Sigma-Aldrich) for 20 min to detect inclusions and with 4′,6-diamidine-2′-phenylindole dihydrochloride (DAPI, Sigma-Aldrich) to identify apoptotic cells. Cells were analysed using the IN Cell Analyzer 2000 (GE Healthcare) as described in [69].

Neurite length of differentiated SH-SY5Y cells was assessed using NeuronJ based on β-tubulin staining and 150–200 cells were counted per condition per experiment. In the conditions without selection medium, a co-staining with eGFP was performed in order to only include transduced cells in the analysis.

### Immunocytochemistry

After a wash step with PBS, cells were fixed with 4% paraformaldehyde for 15 min, followed by another PBS wash step. Cells were permeabilized for 5 min with PBS + 0,1% Triton X-100 (PBST), followed by a 30 min blocking step with 10% goat serum (Dako cytomation) in PBS. Primary antibody was incubated for 2 h in PBS and unbound antibody was removed by three washes with PBS, followed by incubation with secondary antibody (Alexa Fluor 488/555 conjugated antibody, Invitrogen) and three times wash with PBS. Coverslips were mounted on a microscope slide with Mowiol (Sigma-Aldrich) supplemented with DAPI. Visualization and quantification were performed using a laser scanning microscope (LSM 510 META, Carl Zeiss) for neurite outgrowth experiments and TUNEL assays and Zeiss Axio Imager Z.1 for imaging of the LRRK2 distribution pattern.

### Cell lysis and Western blot analysis

Cells were rinsed in PBS and lysed in lysis buffer (Tris 20 mM pH 7.5, NaCl 150 mM, EDTA 1 mM, Triton 1%, glycerol 10%, protease inhibitor cocktail (Sigma-Aldrich) and phosphatase inhibitor (PhosStop, Sigma-Aldrich)). Cell lysates were cleared by centrifugation at 14000 ***g*** for 10 min. Protein content of cell lysates was determined using the bicinchoninic acid (BCA) protein determination assay (Pierce Biotechnology). Total protein extract was resolved by electrophoresis on a NuPage 3-8% tris-acetate gradient gel (Invitrogen) or 3–8% Criterion XT Tris-Acetate gel (Bio-Rad). Separated proteins were transferred to a polyvinylidene fluoride membrane (Bio-Rad) and aspecific binding sites were blocked for 30 min in PBST supplemented with 5% non-fat milk. After overnight incubation at 4°C with primary antibody, blots were washed three times with PBST. After incubation with the appropriate secondary antibody (Dako), blots were again washed as mentioned before. Bands were visualized using enhanced chemiluminescence (Amersham Pharmacia Biotech) and a cooled CCD camera (Fujifilm Las-3000). Densitometric analyses were performed using Aida Analyzer.

### QPCR

LRRK2 mRNA levels of overexpressed LRRK2 were determined using RT-QPCR. Four μg of total RNA was reverse transcribed using the High-Capacity cDNA Archive kit (Applied Biosystems). Five ng cDNA were subsequently used for QPCR analysis with the iQ5 Multicolor RT-PCR detection system (BioRad). The set was directed against WPRE (woodchuck hepatitis virus post-transcriptional regulatory element), a LV-specific transgene. Fw: 5′-CCGTTGTCAGGCAACGTG-3′, Rev: 5′-AGCTGACAGGTGGTGGCAAT-3′, probe:5′-FAM-TGCTGACGCAACCCCCATCGGT-Tamra-3′. WPRE mRNA levels were normalized to β -actin mRNA levels.

### Stereotactic injections and perfusion

Housing and handling of mice were done in compliance with national guidelines; all animal procedures used were approved by the Bioethical Committee of the KU Leuven (Belgium). Eight-week-old female C57BL/6 mice were used for *in vivo* neurite length analysis. The animals were anaesthetized and placed in a stereotactic head frame. After making a midline incision of the scalp, a burr hole was drilled in the appropriate location at both sites of the skull using bregma as reference. Following coordinates were used for mouse striatum: anteroposterior 0.5 mm; lateral 2.0 mm; dorsoventral 4.1 and 3.1 mm. Two times 2 μl was injected in mouse striatum at a rate of 0.25 µl/min with a 30-gauge needle on a 10 μl Hamilton syringe. After the injection, the needle was left in place for an additional 5 min before being slowly withdrawn from the brain. Two weeks later, animals were deeply anesthetized and transcardially perfused with saline.

### Immunohistochemistry and analysis

About 50 µm-thick coronal brain sections were cut with a microtome (HM650V, Microm) and stored at 4°C in PBS with 0.1% sodium azide. Striatal sections were incubated overnight with an in-house anti-eGFP antibody (1/10000) diluted in PBST, 10% sodium azide and 10% goat serum. After three PBST rinses, sections were incubated for 2 h at room temperature with goat anti-rabbit IgG-Alexa 488 (diluted 1: 500, Thermo Fisher Scientific). Sections were again rinsed with PBST and mounted on microscope slides with polyvinyl alcohol (Mowiol; Merck). Neurite length of medium spiny neurons was assessed on Z-stacked confocal images (LSM 510 META, Carl Zeiss) using NeuronJ.

### Statistical analysis

Statistical significance was assessed using a one-way ANOVA with Dunnett test or nonparametric Kruskal–Wallis test using GraphPad software. Normality was tested with a D’Agostino–Pearson normality test. Differences were considered significant when *P*<0.05. Data are expressed as median or means ± SEM.

## Results

### LRRK2 and cell viability

**Figure 1:**
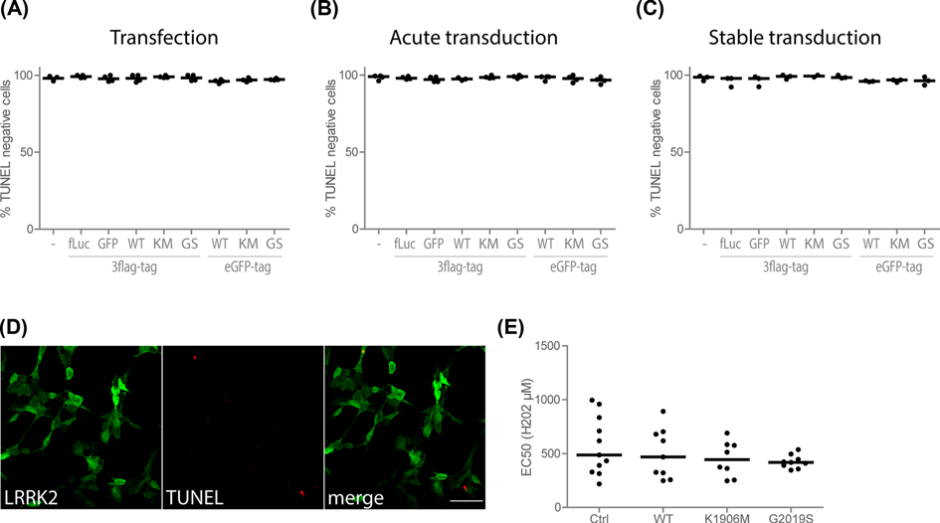
LRRK2 overexpression does not induce cell toxicity in SH-SY5Y cells (**A**-**C**) Percentage of viable cells determined with a TUNEL assay in SH-SY5Y cells transfected with LRRK2 (**A**), 3 days after lentiviral vector transduction (**B**) or 1 month after lentiviral vector transduction (**C**)**.** (**D**) TUNEL assay of SH-SY5Y cells with stable overexpression of 3flag-LRRK2; scale bar is 50 μm. (**E**) EC50 values of H_2_O_2_-induced cell death in SH-SY5Y cell lines selected for 3flag-LRRK2 overexpression, measured using an LDH assay. *N*≥3 independent experiments, for TUNEL experiments: 100–150 cells were analyzed per experiment, all graphs represent the median. Statistical significance was assessed with a Kruskal–Walllis test for (**A–C**) and a one-way ANOVA with Dunnett’s test for (**E**).

Cellular toxicity after overexpression of LRRK2 has been reported by several groups [8–13], but could not be confirmed by others [8,14–17]. Possible reasons for these discrepancies could be the use of different tags and/or the overexpression method. Therefore, we generated SH-SY5Y cell lines overexpressing eGFP- or 3flag-tagged LRRK2 variants by means of transfection, ‘acute’ transduction or ‘stable’ transduction. ‘Acute’ transduction experiments were performed 3 days after lentiviral (LV) vector-mediated transduction. ‘Stable’ transduction was obtained by transduction of cells with LRRK2 LV vectors followed by antibiotic selection. Experiments with these cells were performed at least three passages after selection and expression was confirmed by immunoblotting (Supplementary Figure S1). However, in none of the conditions we could observe adverse effects on cell viability using a Terminal deoxynucleotidyl transferase (TdT) dUTP Nick-End Labeling (TUNEL) assay (Figure 1A–D). To assess whether LRRK2 overexpression affects the sensitivity of cells to oxidative stress, cells with stable overexpression of LRRK2 WT, K1906M or G2019S were treated with a range of H_2_O_2_ concentrations. Cytotoxicity assays based on LDH release revealed that none of the LRRK2 variants increased the sensitivity to oxidative stress (Figure 1E).

### LRRK2 and skein-like structures

**Figure 2:**
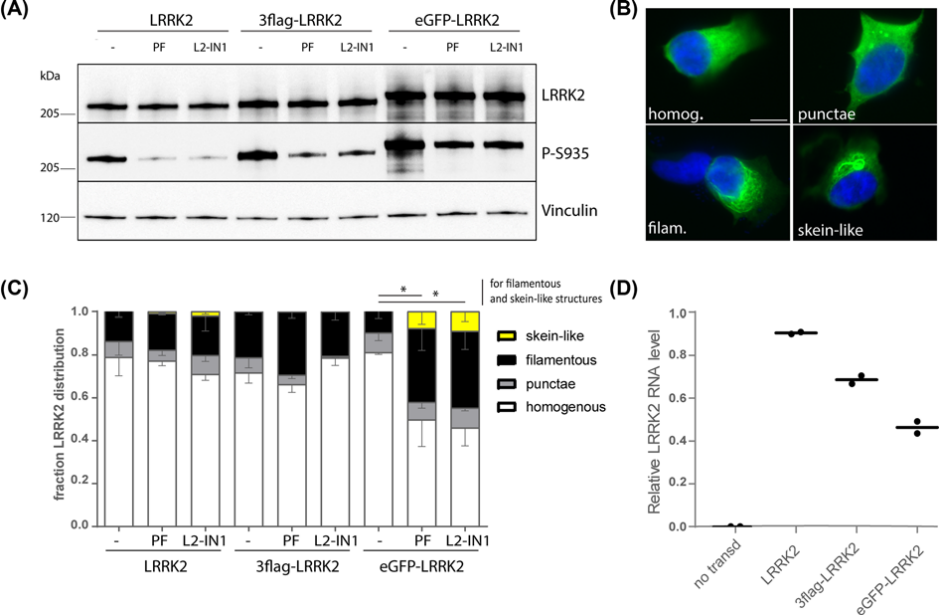
Inhibitor-induced formation of skein-like structures is expression level and/or expression construct dependent (**A**) Immunoblotting of cell extracts from SH-SY5Y cells stably overexpressing LRRK2, 3flag-LRRK2 or eGFP-LRRK2 treated for 2 h with LRRK2-IN1 or PF-06447475. (**B**) Immunocytochemistry for LRRK2 of cells described in (**A**) illustrating a homogenous or filamentous distribution or the presence of punctae or skein-like structures; scale bar is 6 μm. (**C**) Fraction of the cell population presenting the four different distribution types shown in (**B**). *N* ≥ 3 with at least 100 cells per condition per repeat, bars show mean with SEM. (**D**) Graph representing the median relative LRRK2 transgene RNA levels determined by QPCR in the three SH-SY5Y overexpressing cell lines used in (**A–C**). Transgene mRNA levels were normalized to β-actin mRNA levels. Statistical significance was assessed with a one-way ANOVA with Sidak’s multiple comparisons test for filamentous structures and a Kruskal–Walllis test with Dunn’s multiple comparisons test for skein-like structures; *P<0.05

The formation of skein-like structures upon LRRK2 kinase inhibition has been shown by different groups [25,27,31–34,70] using different LRRK2 kinase inhibitors on tagged overexpressed LRRK2. To investigate whether the used tag can influence this phenotype, SH-SY5Y cells with stable overexpression of 3flag-, eGFP- and untagged LRRK2 were treated for 2 h with 1 μM LRRK2-IN1 or 150 nM PF-06447475. Immunoblotting confirmed LRRK2 expression and dephosphorylation of S935 after compound treatment (Figure 2A). To minimize the potential bias by differences in expression level, we aimed to generate cell lines with comparable LRRK2 protein levels. Therefore, a lower titer of eGFP-LRRK2 LV vector, and to a lesser extent 3flag-LRRK2 LV vector was used, compared with untagged LRRK2. This is also reflected in the lower mRNA levels of eGFP and 3flag-tagged LRRK2 (Figure 2D). Cells were stained for LRRK2 using an anti-LRRK2 antibody and classified in four distribution patterns: homogeneous, punctae, filamentous or skein-like (Figure 2B). Quantitative analyses revealed that LRRK2 presents predominantly a homogenous distribution with a small fraction localised in punctae and filamentous structures. Skein-like structures were rare and only evident upon LRRK2 kinase inhibition. Interestingly, inhibition of eGFP-LRRK2 resulted in increased relocalisation to filamentous and skein-like structures, which was not the case for untagged and 3flag-tagged LRRK2 (Figure 2C). These findings suggest that LRRK2 relocalization to skein-like structures is related to inhibitor treatment, and enhanced by the presence of an N-terminal eGFP tag.

### LRRK2 and α-synuclein-related toxicity

**Figure 3:**
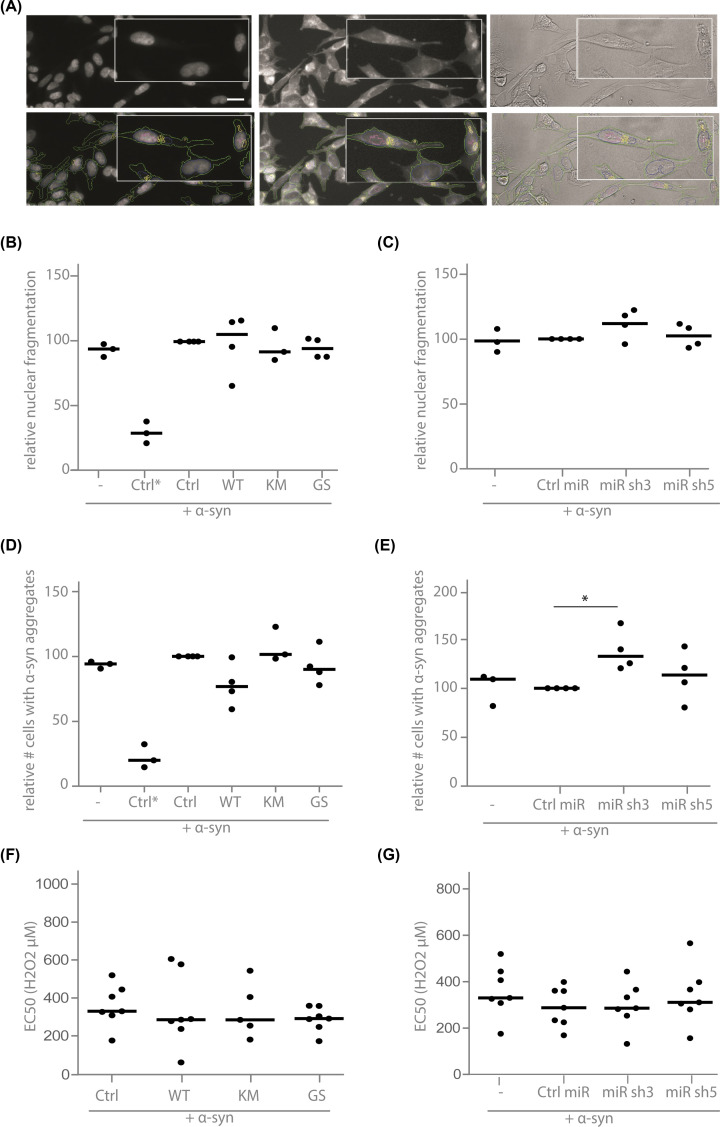
LRRK2 does not play a crucial role in α-synuclein aggregation or toxicity in SH-SY5Y cells (**A**) Representative fluorescent images of SH-SY5Y cells, acquired with an IN cell analyzer (GE Healthcare). SH-SY5Y cells with overexpression of α-synuclein were treated with 100 μM H_2_O_2_ and 5 mM FeCl_2_ for 72 h. Left panels: DAPI staining, middle panels: Thio S staining, right panels: white light images. Representative pictures without (upper panels) and with (lower panels) markers are shown. Blue marker: nucleus, green marker: cytoplasm, yellow marker: Thio S-positive α-synuclein aggregates, pink marker: nuclear condensation/fragmentation to identify apoptotic inclusions. White light images are used to control for correct nucleus and cytoplasm detection; scale bar: 10 μm. High-content analysis of apoptosis (**B** and **C**) or α-synuclein aggregation (**D and E**) in SH-SY5Y cells exposed to FeCl_2_ and H_2_O_2_. Cells overexpress α-synuclein together with LRRK2 overexpression (**B,D**, and **F**) or LRRK2 knockdown (**C,E**, and **G**). Graphs represent the number of cells with fragmented nuclei (**B** and **C**) or number of α-synuclein aggregate-positive cells (**D** and **E**) relative to the control condition. Control conditions include α-synuclein-overexpressing cells without additional transgene but with FeCl_2_ and H_2_O_2_ treatment (-), with overexpression of a non-related protein with hygromycin (overexpression) or blasticidin (knockdown) selection with (Ctrl) or without (Ctrl*) treatment (*N*≥3 independent experiments with for each experiment six wells with in each well approximately 1000 cells analyzed). (**F and G**) EC50 values of H_2_O_2_-induced cell death measured using an LDH assay in SH-SY5Y cell lines selected for LRRK2 overexpression or LRRK2 knockdown in combination with α- synuclein overexpression. (*N*≥5) All graphs represent the median. Statistical significance was assessed with a Kruskal–Walllis test.

Given the evidence for a functional interaction between LRRK2 and α-synuclein [12,17,53–59,71–74], we investigated whether a robust LRRK2 cell culture model could be developed based on this interaction. To this end, we applied an in-house developed automated high-content cellular assay to determine cell toxicity and α-synuclein aggregation in SH-SY5Y cells overexpressing α-synuclein, as previously described [63,69]. We generated an α-synuclein cell line [63] with overexpression of WT, K1906M or G2019S LRRK2 or with LRRK2 knock down (Supplementary Figure S2). Assessment of apoptosis and aggregate formation was based on a Thioflavin S staining [63,69] (Figure 3A). All control conditions are α-synuclein-overexpressing cells and include the following: cells without additional transgene but with FeCl_2_ and H_2_O_2_ treatment (-), with overexpression of a non-related protein with hygromycin (overexpression) or blasticidin (knockdown) selection with (Ctrl) or without (Ctrl*) treatment. This revealed that neither LRRK2 overexpression nor LRRK2 knockdown affects cell toxicity (Figure 3B,C) induced by α-synuclein in this cell culture model. Although the number of cells with α-synuclein aggregates was not affected by overexpression of LRRK2 (Figure 3D), an increase in number of cells with aggregates could be observed in LRRK2 knockdown conditions (Figure 3E). However, this increase could not be replicated using an alternative short hairpin sequence against LRRK2 (miR sh5), despite the stronger reduction in LRRK2 protein levels compared with the miR sh3 sequence (Supplementary Figure S2B). In addition, the sensitivity of these cells to oxidative stress, determined by LDH release, was not different from the control cell line (Figure 3F,G).

### LRRK2 and neurite outgrowth

**Figure 4:**
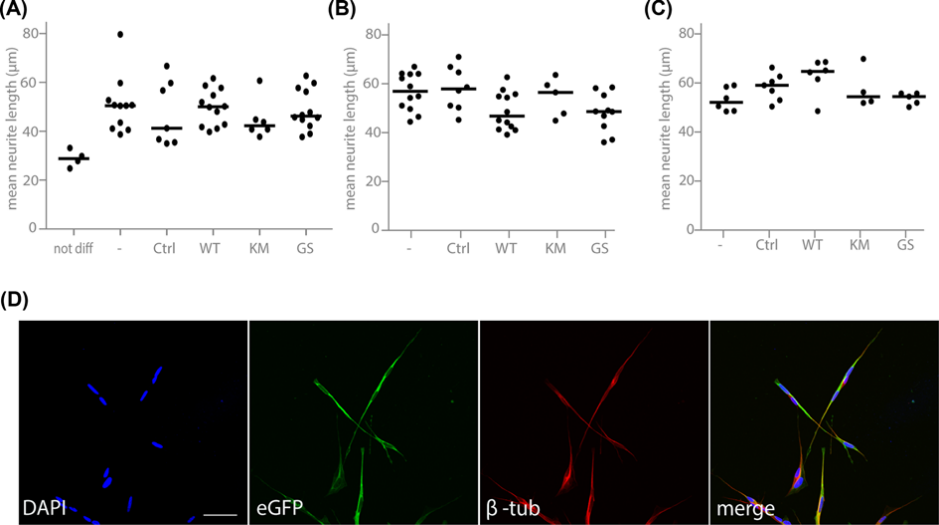
Overexpression of pathogenic LRRK2 does not impair neurite outgrowth or maintenance in SH-SY5Y cells (**A–C**) Graphs representing mean neurite length of RA-differentiated SH-SY5Y cells overexpressing LRRK2. The different conditions are: cells transduced with LV vector 3 days before the start of RA treatment (**A**), cells with stable LRRK2 expression using an antibiotic marker (**B**), or cells transduced with LRRK2 LV vector at the end of the differentiation protocol (**C**). ‘-‘ indicates cells without modification, ‘Ctrl’ is eGFP overexpression. Bars represent the median with *N*≥4 independent experiments and 150–200 cells counted per experiment. Statistical significance was assessed with a Kruskal–Walllis test. (**D**) Illustration of SH-SY5Y cells transduced with lentiviral vectors encoding eGFP-LRRK2 G2019S followed by RA differentiation. The left panel shows nuclear staining, the middle two panels show immunocytochemical staining for eGFP or β-tubulin and a merged picture is shown on the right; scale bar is 50 μm.

Given that pathogenic LRRK2 variants have been reported to reduce neurite length [9–12,15,35–45], we assessed neurite length in differentiated SH-SY5Y cells overexpressing LRRK2. Different LRRK2 overexpression protocols were set up to assess neurite outgrowth, maintenance and retraction. Differentiation was mediated by retinoic acid under following conditions: differentiation of cell lines 3 days after transduction (Figure 4A), differentiation of cell lines with stable, selected expression of LRRK2 variants with LRRK2 LV vectors (Figure 4B) and transduction of cell lines at the end stage of the differentiation protocol (Figure 4C). Neurite length measurements based on β-tubulin staining revealed that overexpression of LRRK2 did not affect neurite outgrowth or did not induce neurite retraction in the conditions tested (Figure 4).

**Figure 5:**
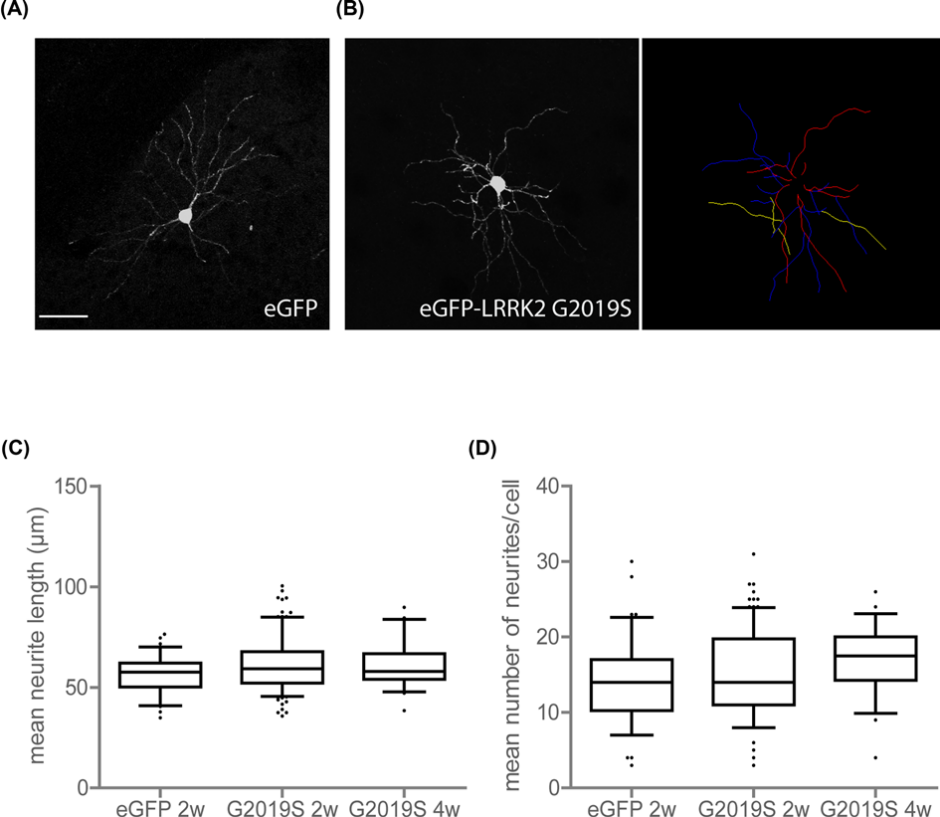
*In vivo* neurite maintenance of MSNs is not affected in conditions of overexpressed pathogenic LRRK2 (**A and B**) Illustration of a striatal medium spiny neuron overexpressing eGFP (**A**) or LRRK2 G2019S with illustration of quantification of length and number per neurite branch analysed using NeuronJ (**B**); scale bar is 50 μm. Mean neurite length of all neurites per cell (**C**) and mean number of neurites per cell (**D**) are quantified. Numbers used for quantification: eGFP 2w: 3 animals, 53 cells in total, LRRK2 G2019S 2w: 5 animals, 86 cells in total, LRRK2 G2019S 4w: 2 animals, 22 cells in total. Statistical significance was assessed with a Kruskal–Walllis test in (**C**) and a one-way ANOVA in (**D**).

Finally, to investigate the effect of pathogenic LRRK2 *in vivo*, we injected LV vectors encoding LRRK2 G2019S into mouse striatum. Neurite length and neurite number of transduced medium spiny neurons (MSNs) were assessed until the quaternary branch. Analysis at 2 and 4 weeks after transduction revealed that overexpression of LRRK2 G2019S did not impair the maintenance of neurite length and organization in the conditions tested (Figure 5).

## Discussion

The assumption that a gain of (toxic) function underlies the pathogenic mechanism of LRRK2 mutations has incited many groups to overexpress LRRK2 variants in cell culture, in order to generate a representative cellular PD model and obtain insight in the mechanisms by which LRRK2 mutations can induce neurodegeneration. With the present study, we set out to identify potential biological and technical parameters, which could explain the observed variation in different described (mutant) LRRK2 phenotypes. Unexpectedly, in the different experimental conditions tested in this study, overexpression of WT or mutant LRRK2 or knock down did not significantly affect cell viability, α-synuclein-induced toxicity or neurite length.

An extensive comparison of the mode of gene transfer, duration of overexpression and the use of different epitope tags, revealed that full-length LRRK2 overexpression per se is not sufficient to elicit cellular toxicity or to affect cellular sensitivity to oxidative stress. This is further supported by the observation that cell lines with stable overexpression of mutant LRRK2 can be generated [16,29,75,76] and cultured for up to 4 months without losing LRRK2 expression (data not shown). Although initial reports suggested a direct link between overexpressed mutant LRRK2 and cytotoxicity in cell culture, it has now become clear that toxic effects of mutant LRRK2 overexpression on cell viability are not always observed [8,14,15,17,77] and are highly variable [8]. This implies that LRRK2-mediated cell death is dependent on additional factors which might include the cell type used [16,78], the level of LRRK2 overexpression [12,77] and the levels of α-synuclein expression [12,17]. Similarly, *in vivo* studies have revealed that overexpression of pathogenic LRRK2 variants in rodents does not necessarily lead to neuronal cell death. Indeed, none of the reported LRRK2 transgenic rodent models expressing physiological levels of LRRK2 nor the BAC strains, which display an expression pattern similar to the endogenous protein, display neurodegeneration [79–84]. Neuronal loss has been reported in LRRK2 transgenic mice with high overexpression levels of mutant LRRK2 and at advanced age [82,85]. However, this phenotype is not consistent for all LRRK2 transgenic mice [40,82,86], or rats [82] and seems to correlate with the degree of overexpression [82]. In analogy, acute expression of LRRK2 G2019S after stereotactic injection of adenoviral or herpes simplex viral vectors in the striatum can cause dopaminergic cell death [82], provided that sufficiently high LRRK2 overexpression levels are reached [87].

The presence of LRRK2-positive accumulations has been frequently described in cells after overexpression of (mutant) LRRK2. Comparison of different LRRK2 variants suggested that the formation of these accumulations is closely related to the phosphorylation status of LRRK2 at S910 and S935 and thus to its 14-3-3 binding [29]. In cell lines with overexpression of dephosphorylated LRRK2 mutants [19,27], a different type of LRRK2 accumulations has been described as fibrillary structures, or so-called skein-like structures. Intriguingly, these structures were also observed in conditions of LRRK2 kinase inhibition, which leads to dephosphorylation at the same serines [25,27,32–34,70]. Previously, we showed that inhibition of protein phosphatase 1, the phosphatase acting on LRRK2 S910 and S935, can prevent LRRK2-IN1–induced LRRK2 dephosphorylation and formation of skein-like structures [27], which corroborates the link with the LRRK2 phosphorylation status. Here, we discriminate between filamentous structures and skein-like structures (Figure 2B). For most of the conditions tested, skein-like structures were very rare, only detectable after kinase inhibition and predominantly present for LRRK2 tagged to eGFP. The observation that filamentous structures were significantly more abundant during kinase inhibition in eGFP-tagged LRRK2 cell lines, but not the other cell lines, is in agreement with another study where eGFP-tagged but not myc-tagged LRRK2 was reported to form aggregates [88]. The choice of tag should therefore be well-considered, taking into account that fusion to eGFP results in a strong increase in protein stability, which is most likely related to the long half-life of eGFP [89]. Taken together, a cellular model, based on LRRK2 inclusions does not appear recommendable given the similar phenotype with pathogenic LRRK2 variants and after LRRK2 kinase inhibition. In addition, these inclusions do not appear to correlate to LRRK2-induced toxicity [12] and have not yet been reported for endogenous LRRK2. Therefore, the exact nature as well as the relevance of these LRRK2 accumulations is still unclear.

Growing evidence suggests a functional interaction between LRRK2 and α-synuclein, with LRRK2 as a modifier of α-synuclein-mediated toxicity. *In vitro* and *in vivo* studies indicate that co-expression of both proteins causes enhanced cellular toxicity [17,53,72]. G2019S LRRK2 was also reported to enhance α-synuclein accumulation/aggregation in cell lines [17], primary neurons, rodent brain [59] and LRRK2 G2019S iPS cell-derived neurons [12,58]. In addition, patient studies revealed that genetic variants of the SNCA gene can lower the age of onset of LRRK2-associated PD [90]. A link between the two proteins was further corroborated by the finding that α-synuclein-induced toxicity is reduced in LRRK2 KO mice or after treatment with LRRK2 kinase inhibitor [53,54,72] or LRRK2 antisense oligonucleotides [91]. However, this link could not be confirmed by several other *in vivo* studies, suggesting that the effect of LRRK2 on α-synuclein-induced toxicity in double transgenic animals (α-synuclein + LRRK2 G2019S or + LRRK2 KO/kinase inhibition) depends on the model used [53,54,72,86,92–95] and the overexpression levels of LRRK2 [72]. Also here, we did not observe a robust effect of LRRK2 on α-synuclein-induced toxicity and aggregate formation in the cell lines tested. Future studies will need to provide insight in the exact nature of the LRRK2- α-synuclein interaction in PD in order to identify modifiers of disease mechanisms and thus potential new targets for therapeutic strategies.

The effect of LRRK2 on neurite growth/branching has been proposed as a more specific phenotype in line with the numerous reported links between LRRK2 and the cytoskeleton [96]. Nevertheless, in our hands LRRK2 overexpression did not significantly induce neurite retraction or impaired neurite outgrowth in cell culture and *in vivo*, which is in agreement with other reports [14,15,30,46,97,98]. Cell-type and neurite-specific effects might be in place as decreased dendrite and axon length was observed in primary cortical neurons, but increased dendrite length and no effect on axons in dopaminergic cultures [15]. In the striatum, LRRK2 is suggested to be equally expressed in direct and indirect pathway neurons [99]. Interestingly, although no effect on dendritic length could be observed, expression of LRRK2 G2019S was shown to induce an increase in action potential-dependent activity, compared with LRRK2 WT, with similar effects in both direct and indirect pathway MSNs [98]. Here, we did not discriminate between D1R- and D2R-type MSNs, hence potential cell-type-specific effects on neurite length cannot be excluded.

In addition, the level of LRRK2 overexpression is a likely contributing factor, since overexpression of G2019S LRRK2 reduced neurite length, while primary neurons from a G2019S knockin mouse did not display impaired neurite outgrowth in the same experimental setup [46]. Still, the LRRK2-associated neurite outgrowth phenotype appears to be mutant-specific since high overexpression levels of WT LRRK2 did not affect neurite morphology in this setup [46]. Finally, discrepancies in the field might also be attributed to the stage of neuronal development [14,28,46] and the choice of growth substrate [14].

Altogether, our data clearly show that commonly used cellular LRRK2 phenotypes such as cell death, LRRK2 inclusion formation, effects on α-synuclein-mediated toxicity and a disturbed neurite network, depend on secondary, yet unknown, variables. These factors, which may be both environmental and genetic, might reflect the incomplete penetrance [100,101] and highly variable neuropathology [102] observed in patients with LRRK2 mutations. Indeed, genetic variations in the SNCA [90,103], MAPT [104] or BDNF [105] gene in mutant LRRK2 carriers can affect the age of onset or the risk to develop PD. Therefore, identification of these modifying factors, which might explain inconsistencies in the field, will be crucial to generate an adequate cellular LRRK2 PD model, which (at least partly) recapitulates the pathological mechanisms in patients and thus provides a reliable platform for new therapeutic strategies.

## Supplementary Material

Supplementary Figures S1-S2

## Abbreviations

BDNF: brain-derived neurotrophic factor
DAPI: 4′,6-diamidine-2′-phenylindole dihydrochloride
FCS: fetal calf serum
L2-IN1: LRRK2 kinase inhibitor-1
LDH: lactate dehydrogenase
LRRK2: leucine-rich repeat kinase 2
LV: lentiviral
MAPT: microtubule-associated protein tau
PAK6: P21 activated kinase 6
PBS-T: PBS + 0,1% Triton X-100
PD: Parkinson’s disease
SNCA: alpha-synuclein
TUNEL: terminal deoxynucleotidyl transferase dUTP nick end labeling
WT: wild-type
