# Supplementary material for: Pathogenic LRRK2 requires secondary factors to induce cellular toxicity
Source: Biosci Rep. 2020 Oct 14;40(10):BSR20202225. doi: 10.1042/BSR20202225 (PMC7560525; doi:10.1042/BSR20202225)
Supplement: Supplementary Figures S1-S2 [file BSR-2020-2225_supp.pdf]

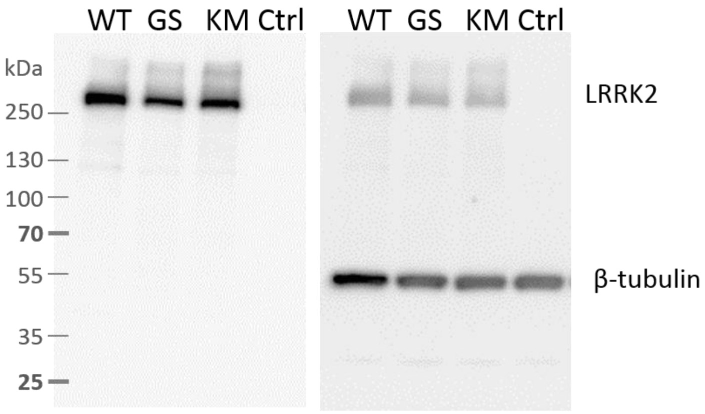

WT GS KM Ctrl
WT GS KM Ctrl
kDa
250
130
100
70
55
35
25
LRRK2
β-tubulin

Figure S1 LRRK2 overexpression in stable selected SH-SY5Y cell lines. Immunoblotting showing overexpression of 3flag-LRRK2 WT, G2019S or K1906M or eGFP as a control. The full length blot with LRRK2 detection is shown on the left, subsequent β-tubulin detection on the right.

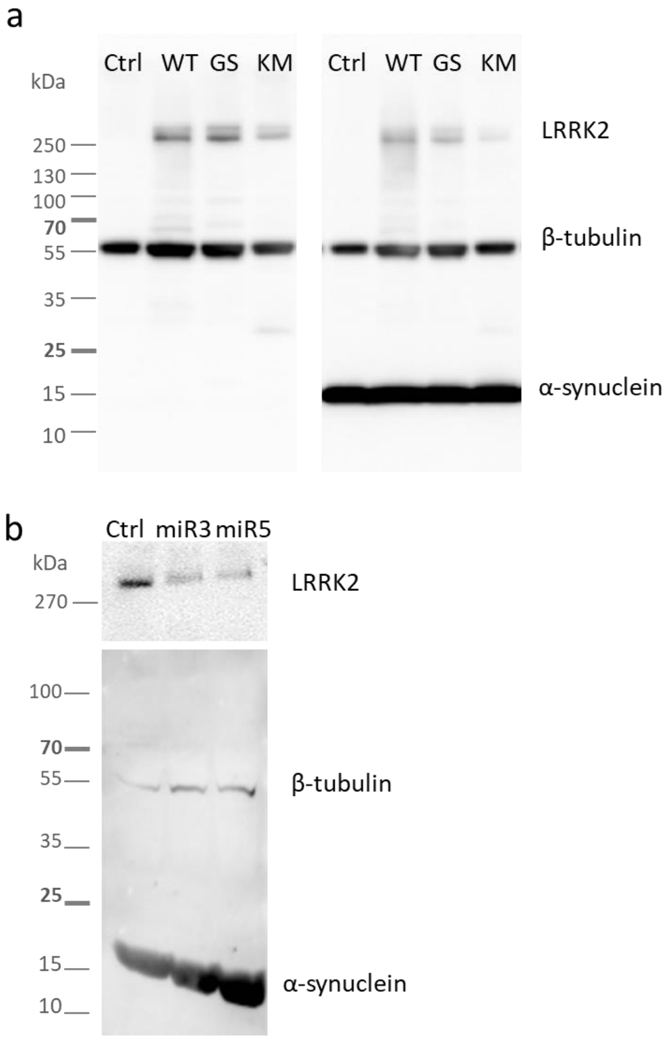
a
Ctrl WT GS KM
Ctrl WT GS KM
kDa
250
130
100
70
55
35
25
15
10
LRRK2
β-tubulin
α-synuclein
b
Ctrl miR3 miR5
kDa
270
LRRK2
100
70
55
35
25
15
10
β-tubulin
α-synuclein

Figure S2 Alpha-synuclein overexpression combined with LRRK2 overexpression or knock down in stable double selected SH-SY5Y cell lines. a. Immunoblotting showing overexpression of 3flag-LRRK2 WT, G2019S or K1906M or eGFP as a control. The full length blot with LRRK2 and β-tubulin detection is shown on the left, subsequent α-synuclein detection on the right. **b.** LRRK2 knock down using two different miRNA-based short hairpin sequences. A short hairpin sequence against firefly luciferase is included as negative control.
